# Cardiovascular Health Does Not Change Following High-Intensity Interval Training in Women with Polycystic Ovary Syndrome

**DOI:** 10.3390/jcm11061626

**Published:** 2022-03-15

**Authors:** Ida Almenning Kiel, Helen Jones, Sofie Lionett, Ragnhild Røsbjørgen, Stian Lydersen, Eszter Vanky, Trine Moholdt

**Affiliations:** 1 Department of Circulation and Medical Imaging, Faculty of Medicine and Health Sciences, Norwegian University of Science and Technology, 7491 Trondheim, Norway; ida.almenning@ntnu.no (I.A.K.); sofielionett@gmail.com (S.L.); ragnhild.rosbjorgen@ntnu.no (R.R.); 2 Department of Obstetrics and Gynecology, St. Olav’s Hospital, Trondheim University Hospital, 7030 Trondheim, Norway; eszter.vanky@ntnu.no; 3 Research Institute for Sport and Exercise Sciences, Liverpool John Moores University, Liverpool L3 5UX, UK; h.jones1@ljmu.ac.uk; 4 Regional Centre for Child and Youth Mental Health and Child Welfare, Department of Mental Health, Norwegian University of Science and Technology, 7491 Trondheim, Norway; stian.lydersen@ntnu.no; 5 Department of Clinical and Molecular Medicine, Faculty of Medicine and Health Sciences, Norwegian University of Science and Technology, 7491 Trondheim, Norway

**Keywords:** PCOS, exercise, cardiovascular disease, HIIT, flow-mediated endothelial function, matrix metalloproteinase-9

## Abstract

Introduction: polycystic ovary syndrome (PCOS) is associated with cardiovascular disease (CVD) risk factors. First-line therapy for PCOS is lifestyle changes including exercise. We compared CVD risk factors between women with and without PCOS and examined the responses to high-intensity interval training (HIIT). Methods: women with PCOS were randomized to HIIT (*n* = 41) or a non-exercise control group (*n* = 23) for 16 weeks. Women without PCOS (*n* = 15) were age- and BMI-matched to participants with PCOS and completed 16 weeks of HIIT. CVD markers included blood pressure, heart rate, flow mediated dilatation (FMD), carotid intima-media thickness (IMT), and circulating concentrations of lipids, glucose, insulin, and matrix metalloproteinase-9 (MMP-9). Results: resting heart rate was higher in women with PCOS than without PCOS (*p* =0.011) and was reduced after HIIT in women with PCOS (−2.8 beats/min, 95% CI: −5.4, −0.2, *p* = 0.037). FMD was not significantly different between women with PCOS (5.5%, SD 4.1) and those without PCOS (8.2%, SD 3.9) at baseline. HIIT reduced time-to-peak dilatation of the brachial artery in women with PCOS compared with women without PCOS (−55 s, 95% CI: −96, −13, *p* = 0.012). Conclusions: we found little difference in CVD risk factors between women with and without PCOS at baseline, but some indications of endothelial dysfunction in women with PCOS.

## 1. Introduction

Polycystic ovary syndrome (PCOS) is the most common endocrine disorder in women of reproductive age. PCOS is associated with multiple cardiovascular disease (CVD) risk factors already present in the pre-menopausal age in this clinical population [1,2]. Early detection of CVD risk and novel CVD biomarkers are, thus, clinically relevant among women with PCOS. 

Endothelial dysfunction measured as flow mediated dilatation (FMD) and carotid intima-media thickness (IMT) are surrogate markers of CVD risk [3,4] and represent early signs of future cardiovascular events [4,5,6]. Earlier studies have suggested that women with PCOS have reduced endothelial function [7], independent of body mass index (BMI) and fat distribution [7,8], and increased IMT compared with women without PCOS [9]. Matrix metalloproteinase-9 (MMP-9) is a zinc-dependent enzyme involved in the degradation of extracellular matrix [10] and has been linked to vascular remodeling and atherosclerosis [11], as well as ovarian dysfunction [12]. Increased circulating levels of MMP-9 have been reported in women with PCOS [10,13,14]. MMP-9 is suggested to play a key role in the pathogenesis of PCOS [15,16] and the increased CVD risk in these women [10,14]. 

Lifestyle modification, including exercise training, is the first-line therapy for women with PCOS, with studies demonstrating positive effects of moderate intensity exercise training on endothelial function [17,18,19]. However, there is limited evidence for effects of other exercise modalities on cardiovascular biomarkers, endothelial function, and CVD risk factors in PCOS [20]. In a pilot randomized controlled trial (RCT), we investigated the effect of 10 weeks of high-intensity interval training (HIIT) versus strength training, compared with a no-exercise control group. We reported no statistically significant between-group difference in FMD [19], but a significant within-group increase in FMD after HIIT. No prior study has investigated the effect of exercise training on circulating MMP-9 in women with PCOS. Moderate intensity endurance training reduced this CVD risk marker in individuals with type 2 diabetes [21], indicating modification of MMP-9 as one of the underlying mechanisms of exercise-induced cardiovascular protection. 

Our aims were, firstly, to compare cardiovascular risk markers (blood pressure, resting heart rate, endothelial function, IMT, and circulating concentrations of lipids, glucose, insulin, and MMP-9) between women with and without PCOS, and secondly, to assess the effect of 16 weeks of HIIT on these markers in both groups of women. 

## 2. Materials and Methods

### 2.1. Study Design 

The study is a secondary analysis of a two-center RCT in women with PCOS (the IMPROV-IT trial) conducted at The Norwegian University of Science and Technology (NTNU) in Trondheim, Norway, and The Australian Catholic University (ACU) in Melbourne, Australia, and a case-control study including women without PCOS conducted at NTNU in Norway. The study protocol and primary results from the IMPROV-IT trial has been published elsewhere [22,23]. Results on other secondary outcomes from the IMPROV-IT trial has also been published previously [24,25].

Women with PCOS were randomly allocated in a 1:1:1 manner to 16 weeks of semi-supervised low-volume HIIT, or high-volume HIIT (PCOS HIIT), or a non-exercise control group (PCOS Non-ex) after stratification for BMI < or ≥ 27 kg/m^2^ and study center. In the case-control trial, women without PCOS were individually matched by age (±5 years) and BMI (±2 kg/m^2^) to women with PCOS in the IMPROV-IT trial. Women without PCOS were randomly allocated in a 1:1 manner to 16 weeks of semi-supervised low-volume HIIT or high-volume HIIT (Non-PCOS HIIT) after stratification for BMI < or ≥ 27 kg/m^2^. A computer random number generator developed and administered at the Faculty of Medicine, Department of Public Health and General Practice, NTNU, Trondheim, Norway, was used in both trials. 

Both trials were approved by the Regional Committee for Medical Research Ethics in Central Norway (2015/468/REK midt and 2016/545 REK midt), and the IMPROV-IT trial was also approved by the ACU Human Research Ethics Committee (2017-260H). Participants received written and oral information and signed an informed consent before inclusion in the trials. Both trials are registered in ClinicalTrials.gov (NCT02419482, NCT02943291).

### 2.2. Participants

To be eligible for inclusion, the women had to be between 18–45 years old. The following exclusion criteria were applied in both trials: regular endurance training (≥2 times/week), ongoing treatment with hormonal contraceptives, insulin sensitizers or drugs known to affect gonadotropin or ovulation (with a three-month wash out period prior to inclusion), on-going pregnancy, breast feeding for the last 24 weeks, cardiovascular disease, or other endocrine disorders. Inclusion criteria in the IMPROV-IT trial were based upon PCOS diagnosed according to the Rotterdam criteria [26], in which a minimum of two out of three criteria must be fulfilled: (1) polycystic ovary morphology (12 or more 2–9 mm follicles or >10 mL in volume in at least one ovary), (2) clinical or biochemical hyperandrogenism (hirsutism, and/or elevated androgens), and/or (3) oligo/amenorrhea. For women without PCOS, the inclusion and exclusion criteria were the same as for women with PCOS, but they had a normal menstrual cycle and no known evidence of hyperandrogenism or polycystic ovary morphology. 

### 2.3. Interventions

The HIIT protocols were semi-supervised during the 16-week intervention period. Previous publications provide a detailed description of the HIIT intervention [22,23]. Briefly, the participants were offered three weekly supervised exercise sessions, but could choose to exercise unsupervised for up to two of the three weekly sessions. During the supervised sessions, the participants walked or ran on a treadmill, whereas unsupervised sessions could also be undertaken outdoors. The HIIT sessions started with a 10-min warm-up and ended with a 3-min cool-down. The high-volume HIIT protocol (HV-HIIT) included four 4-min work bouts at an intensity corresponding to 90–95% of maximal heart rate, interspersed by a 3-min active recovery at ~70% of maximal heart rate. The low-volume HIIT protocol (LV-HIIT) included ten 1-min work-bouts at the maximal intensity the participants could sustain, interspersed by a 1-min low-intensity walking or passive recovery. 

The control group (PCOS Non-Ex) was advised to continue their habitual daily physical activity and diet, but they were informed about the current recommendations of at least 150 min weekly moderate intensity physical activity. Physical activity was estimated from 5-day activity monitoring (Sensewear Armband, APC Cardiovascular, UK) and diet was recorded using a 4-day diet recall, at baseline and after 16 weeks for all groups. 

### 2.4. Outcomes

**Figure 1:**
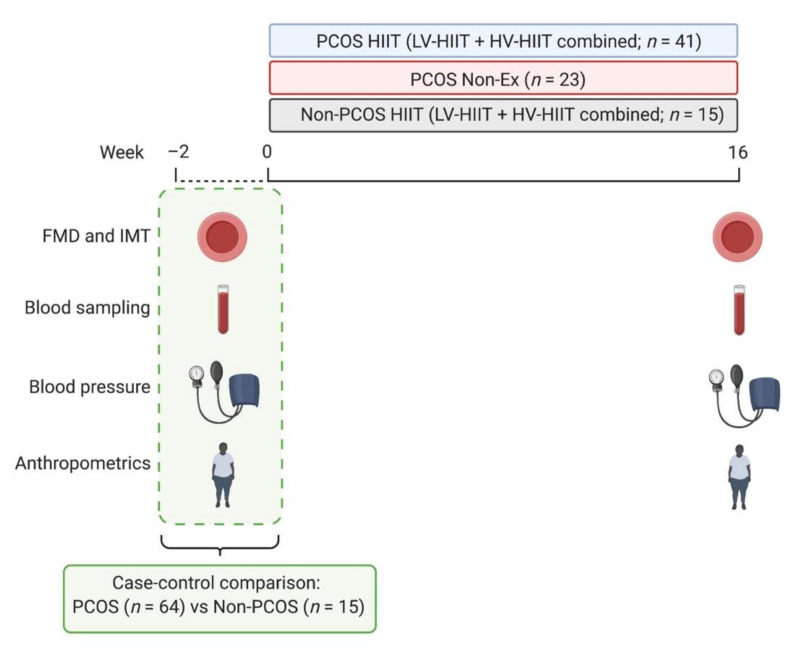
Study protocol. Women without polycystic ovary syndrome (Non-PCOS) were matched for age body mass index (BMI) to women with polycystic ovary syndrome (PCOS). Low-volume high-intensity interval training (LV-HIIT) and high-volume high-intensity interval training (HV-HIIT) were pooled in the analysis for both women with and without PCOS to improve statistical power. FMD = flow-mediated dilatation, IMT = carotid intima-media thickness, Non-Ex = non-exercise control group.

Measurements were undertaken at baseline and after 16 weeks in the follicular phase (day 1–7 in the menstrual cycle) in participants with regular menstrual cycles. Women with oligo/amenorrhea were tested independent of their menstrual cycle. Participants arrived fasted (≥12 h), and with no smoking, caffeine, or exercise allowed for 48 h prior to assessments. Figure 1 displays an overview of the study protocol.

#### 2.4.1. Flow-Mediated Dilatation and Intima-Media Thickness 

Measurements of FMD and IMT were obtained at the same time of day at baseline and after 16 weeks. Participants rested in a supine position for 20 min before assessments. We used a high-resolution B-mode ultrasound machine (GE Vingmed Ultrasound AS, Horten, Norway) with a 12 MHz Doppler probe. The same, trained person obtained all the ultrasound recordings.

For the brachial artery FMD measurements, the ultrasound probe was placed on the upper arm perpendicular to the brachial artery, with a blood pressure cuff placed distal to the brachial artery to produce the stimulus of forearm ischemia. When an optimal image was obtained, the probe was held stable, and the ultrasound parameters were set to optimize B-mode images of the lumen-arterial wall interface. Resting baseline diameter was recorded continuously for 1 min before the cuff was inflated (200–250 mmHg) for 5 min. Diameter was resumed 30 sec prior to cuff deflation and continuously recorded for 3 min after deflation. We did not include shear rate data in these measurements since we experienced technical difficulties when saving the Doppler assessments of velocity. We allometrically scaled the FMD data as described by Atkinson et al. [27]. This allometric scaling is more accurate for scaling changes in diameter than simple percentage change [27]. 

For IMT measurements, the ultrasound probe was placed perpendicular to the common carotid artery, 1 cm below the bifurcation on the right side, with the participant’s head hyperextended and turned to the left. Measurements were performed in three angles: transversal, longitudinal, and anterolateral. The minimal diameter during the cardiac cycle was measured and IMT was defined as the distance between the intima and media layer on a B-mode image with both the near and far walls clearly visualized with a double-line pattern in longitudinal plane [28]. The average of three measurements on the far wall was calculated and used in the analysis. 

We used an automated edge-detection and wall tracking software (Brachial Analyzer and carotid Analyzer for Research, Medical Imaging Application, LLC, Coralville, IA, USA) for brachial and carotid artery diameter analysis.

#### 2.4.2. Blood Pressure and Circulating Markers 

Blood pressure was measured after 15 min rest using an automatic blood pressure device (IntelliVue MP50, Philips House, Ltd., Dublin, Ireland). Three repeated measurements with 2 min intervals were taken, with the average used in the analysis. 

Fasting venous blood samples were obtained, and concentrations of lipids (total cholesterol, high-density lipoprotein (HDL), and triglycerides) were analyzed with Advia chemistry XPT, Siemens, Erlangen, Germany. Low-density lipoprotein-cholesterol (LDL) was calculated according to the Friedewald formula with LDL cholesterol measured in mmol/L: Total cholesterol—HDL cholesterol—(triglyceride/2.2) [29]. Participants undertook a 2-h oral glucose tolerance test (OGTT; 75 g glucose diluted in 250 mL water) for measurements of glucose and insulin concentrations at 0 (prior to OGTT), 30, 60, 90, and 120 min. Plasma glucose concentrations were analyzed with Roche Moduclar P (Roche, Switzerland). Serum insulin concentrations were analyzed in duplicate with an enzyme-linked immunosorbent assay (ELISA, IBL-International, Hamburg, Germany) using a Dynex-DS2 Two-plate Automated ELISA Processing System (DYNEX Technologies, Chantilly, VA, USA).

Insulin resistance was estimated with the Homeostatic Model Assessment for Insulin Resistance (HOMA-IR); fasting serum insulin (μIU/mL) × fasting plasma glucose (mmol/L) divided by 22.5. We calculated the total area under the curve for glucose and insulin using a baseline of 0 mmol/L, and incremental area under the curve with fasting concentrations as baseline values, using a conversion factor for insulin of 1 µIU/mL = 6.00 pmol/L.

We analyzed MMP-9 in plasma as recommended [30] using ELISA (R&D systems, Inc., Minneapolis), in duplicate using a Dynex-DS2 Two-plate Automated ELISA Processing System (as described above). We chose to exclude samples with hemolysis since the MMP-9 concentrations in these samples were substantially higher than in the samples without hemolysis.

#### 2.4.3. Statistical Analyses 

Results reported here are from a clinical trial (IMPROV-IT), which was designed to determine the effect of HIIT on menstrual frequency during 12 months follow-up in women with PCOS [22]. We did not calculate sample size for the analyses included here since the reported data are secondary outcome measures from the IMPROV-IT trial. Our sample size corresponds to other studies with similar outcomes [7,18,21,31]. Since there were no between-group differences for any of the outcome measures between LV-HIIT and HV-HIIT in any group of women, we pooled the two HIIT protocols for both women with and without PCOS to increase statistical power. 

We used linear mixed models with the outcome variable as dependent variable, participant as random factor, and the time and group allocation and their interaction as fixed effects. Baseline values for the participants were adjusted for in the model, as recommended by Twisk et al. [32]. Baseline data are reported as mean and standard deviation (SD), and comparisons within and between groups are reported as estimated means with 95% confidence intervals. The normality of residuals was evaluated by visual inspection of Q-Q plots. In three of the dependent variables (insulin, HOMA-IR, MMP-9) the Q-Q-plot of residuals indicated a slight deviation from normality, and bootstrapping was performed (with bias corrected, accelerated method and 1000 bootstrap samples). The results were similar after bootstrapping. We consider *p*-values < 0.05 as statistically significant. IMB SPSS Statistics 26 (SPSS Inc., Chicago, IL, USA) was used for all statistical analyses.

## 3. Results

Sixty-four women with PCOS and 15 women without PCOS were included in this study. Supplementary Figure S1 outlines the participant CONSORT flow diagram. The distribution of the four PCOS phenotypes was: 30% (*n* = 19) with phenotype A (oligo/amenorrhea + hyperandrogenism + polycystic ovaries), 9% (*n* = 6) with phenotype B (oligo/amenorrhea + hyperandrogenism), 30% (*n* = 19) with phenotype C (hyperandrogenism + polycystic ovaries), and 31% (*n* = 20) with phenotype D (oligo/amenorrhea + polycystic ovaries). The age distribution for women with and without PCOS, respectively, was: 56% (*n* = 36) and 47% (*n* = 7) aged 18–29 years old, 41% (*n* = 26) and 40% (*n* = 6) aged 30–39 years old, and 3% (*n* = 2) and 13% (*n* = 2) aged between 40–45 years old. Ten participants dropped out (PCOS HIIT, *n* = 6; PCOS Non-Ex, *n* = 3; Non-PCOS HIIT, *n* = 1) and five participants became pregnant during the intervention period (PCOS HIIT) and were excluded from analysis. For the FMD and IMT measurements, a subgroup of 39 participants with PCOS and 11 age- and BMI-matched controls without PCOS were included. Four of the participants were habitually using nicotine. 

### 3.1. Baseline Comparisons between Women with and without PCOS 

Table 1 shows baseline characteristics of women with and without PCOS. Women with PCOS had a higher resting heart rate than those without PCOS. There were indications that women with PCOS had lower absolute FMD (mm), lower FMD (%), and longer time-to-peak dilatation compared with the Non-PCOS group, but these differences were not statistically significant (Table 1). There was also a tendency for women with PCOS to have higher circulating insulin levels. Although no statistically significant difference was evident, 43 participants (68%) in the PCOS group versus five participants (42%) in the Non-PCOS group had a HOMA-IR ≥ 2.5.

### 3.2. Cardiovascular Responses to High-Intensity Interval Training 

The PCOS HIIT group exercised on average (SD) 2.2 (0.5) sessions per week, whereas the Non-PCOS HIIT group exercised on average (SD) 2.6 (0.4) sessions per week (*p* = 0.03). We were not able to include exercise training data for seven women in the PCOS HIIT group and one woman in the Non-PCOS HIIT group because they did not register their exercise training sessions in Polar Flow.

Table 2 shows biochemical and vascular outcomes at baseline and after 16 weeks. There was a significant within-group decline in resting heart rate in the PCOS HIIT group (*p* = 0.037). Participants in PCOS Non-Ex had significantly lower body weight (*p* < 0.001), BMI (*p* < 0.001), systolic blood pressure (*p* = 0.001), HDL cholesterol (*p* = 0.010), insulin levels (*p* = 0.026), and HOMA-IR (*p* = 0.032) after 16 weeks compared to baseline, and there was a between-group difference in BMI compared to PCOS-HIIT (*p* = 0.031). MMP-9 did not differ between participants with and without PCOS, and there were no within- or between-group differences in MMP-9 after 16 weeks.

**Figure 2:**
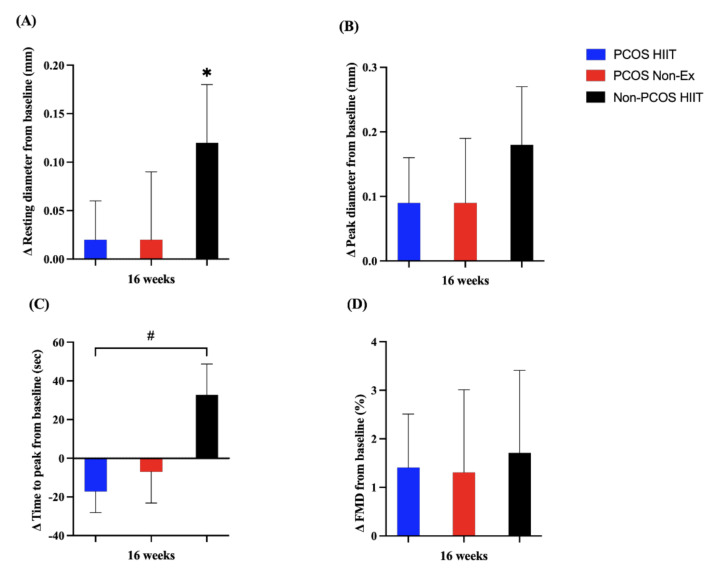
Effects of 16 weeks of high-intensity interval training or non-exercise: (**A**) resting diameter (mm); (**B**) peak diameter (mm); (**C**) time-to-peak (sec); (**D**) FMD (%). The PCOS high-intensity interval training group (PCOS HIIT) is depicted in blue bars, the PCOS Non-exercise group (PCOS Non-Ex) in red bars, and the Non-PCOS high-intensity interval training group (Non-PCOS HIIT) in black bars. The bars and error bars represent estimated means and standard error (SE) based on linear mixed model. ^#^ Between-group difference (*p* = 0.012), * Within-group difference (*p* = 0.038).

Non-PCOS HIIT increased their resting brachial artery diameter from baseline to 16 weeks (0.12 mm, 95% CI: 0.01, 0.24, *p* = 0.038), and there was a tendency for increased peak diameter (0.18 mm, 95% CI: 0.00, 0.36, *p* = 0.046) and time-to-peak (33 s, 95% CI: −1, 67, *p* = 0.055) in this group of women (Figure 2). There was a significant between-group difference in change in time-to-peak dilatation between the PCOS HIIT group and the Non-PCOS HIIT group after 16 weeks of HIIT (55 sec, 95% CI: 13, 96, *p* = 0.012), but not compared with the PCOS Non-Ex group (Figure 2).

## 4. Discussion

In this comparison of cardiovascular health markers in women with PCOS compared with age- and BMI-matched women without PCOS, we report little differences between the two groups of women at baseline. Although not statistically significant, our data indicate that women with PCOS have reduced endothelial function compared to women without PCOS, and our results are comparable with other studies with statistically significant findings [8,33]. There was no effect of a semi-supervised HIIT intervention for 16 weeks on blood pressure, circulating lipids, or MMP-9 in women with PCOS. In women without PCOS, resting artery diameter increased after the HIIT intervention. There was a between-group difference in training response in time-to-peak FMD of the brachial artery, with a decreased time-to-peak dilatation in women with PCOS compared with those without PCOS. 

### 4.1. CVD Risk Factors between Women with and without PCOS

There was no statistically significant difference in FMD between women with and without PCOS in our study, which contrasts previous research [7,8]. Sprung et al. [8] included women with PCOS who presented all three criteria for diagnosis (polycystic ovaries, oligo/amenorrhea, and hyperandrogenism), thus, a more severe PCOS phenotype than our participants and, thereby, a higher risk for CVD [34]. Indeed, a large proportion (61%) of our participants had phenotypes which are considered milder forms of PCOS (phenotype C and D). Obesity, dyslipidemia, and hypertension are more prominent in women with PCOS phenotypes A and B than phenotypes C and D [35], leading to increased CVD risk. 

We found no difference in IMT between women with and without PCOS, which may be explained by similar insulin levels, lipid profiles, and BMI in the two groups. Carotid IMT is reported to be significantly higher among women with PCOS compared with controls, and may be related to higher insulin levels, dyslipidemia, and abdominal obesity reported in women with PCOS [9]. Our low sample size on the IMT measurements may also have affected our results.

We found no difference in plasma MMP-9 concentrations between women with and without PCOS, which is in line with findings from Gomes et al. [36]. In contrast, Lewandowski et al. [14] reported elevated circulating MMP-9 levels in women with PCOS diagnosed with all three criteria (oligo-/amenorrhea, hyperandrogenism, and polycystic ovaries) compared with women without PCOS. Since we used the Rotterdam criteria, not necessitating that all three criteria are fulfilled, we may have included a sample of women with PCOS with a less severe phenotype compared with the study from Lewandowski and colleagues (e.g., our sample had a lower BMI at baseline). A cross-sectional study on infertile women with PCOS showed that circulating MMP-9 concentrations were increased only for those with obesity and PCOS, and that MMP-9 levels were associated with the duration of infertility [13]. Our result could, therefore, potentially be explained by the inclusion of both fertile and infertile women with PCOS, and also of women with a normal BMI. 

### 4.2. The Effects of HIIT on CVD Risk Factors

There is strong scientific evidence for the effect of exercise on CVD risk factors [37], also in women with PCOS [38,39,40]. Studies have demonstrated significant improvement in FMD after moderate-intensity exercise, also in the absence of a change in body composition, [18] and with favorable within-group improvements in other cardiovascular risk factors in women with PCOS [17]. Further, a significant within-group difference in FMD was observed in women with PCOS after 10 weeks of HIIT in our pilot RCT [19]. The adherence to HIIT was higher (~90% of the scheduled sessions) in the pilot RCT. Despite no effects of HIIT on FMD, we found lower time-to-peak dilatation in women with PCOS compared with Non-PCOS after HIIT. We found no difference in IMT after 16 weeks of HIIT in our study. Others have reported a significant decrease in IMT in women with obesity after exercise training (combined aerobic and resistance training) [41,42], as well as in women with PCOS after moderate-intensity exercise [17]. IMT is associated with insulin levels, lipid profile, BMI, and/or abdominal obesity in women with PCOS [9,43], and there was no effect of HIIT on these variables in our study. Furthermore, the women with PCOS in our study had IMT within normal limits at baseline, with no between-group difference compared with the women without PCOS. 

We did not detect an effect of HIIT on circulating MMP-9 levels in any group of women. This finding contrasts with a previous study which showed decreased circulating plasma MMP-9 levels after moderate-intensity exercise training in individuals with type 2 diabetes [21]. 

Lastly, our results indicate that participants with PCOS who were allocated to the control group may have changed their lifestyle during the intervention period, as evident by significant improvements in body weight, BMI, systolic blood pressure, circulating insulin, and HOMA-IR after 16 weeks. We hypothesize that the lack of effects on CVD risk factors in the HIIT groups could be explained by the low adherence to the exercise protocol (37% of the participants adhered to the prescribed exercise protocol). We speculate that the low adherence to HIIT may be due to the long intervention period and that all sessions were identical throughout the entire intervention. As such, we experienced substantially higher adherence (~90%) in our pilot study, which comprised a 10-week HIIT intervention with a combination of different HIIT protocols [19]. 

### 4.3. Strength and Limitations

Strengths of this study include the RCT design, the individually age- and BMI-matching of women with and without PCOS, and the strict inclusion and exclusion criteria. We also acknowledge study limitations. As in other RCTs in which participants are randomly allocated to a lifestyle intervention or a control group, lifestyle modifications in the control group limit the possibility to detect between-group differences. Our results indicate that participants with PCOS who were allocated to the control group may have changed their lifestyle during the intervention period, as evident by significant improvements in several of the CVD risk factors after 16 weeks. The sub-optimal adherence to the prescribed exercise protocols in the intervention groups and low sample size in some of the measured outcomes are other limitations. 

### 4.4. Future Perspectives

Interventions with more motivational support or behavior change techniques should be explored to improve adherence to exercise interventions among women with PCOS. Exercise protocols with more supervision, more variation, and/or a more personalized exercise protocol should be investigated to prevent relapse during lifestyle interventions. It is necessary to further explore the role of MMP-9 as a potential biomarker and risk predictor for CVD in PCOS and the role of MMPs in PCOS pathophysiology.

## 5. Conclusions

Apart from lower resting heart rate and a tendency of lower endothelial function in women with PCOS diagnosed according to the Rotterdam criteria compared with age- and BMI-matched women without PCOS, there were no differences in selected CVD risk factors between these two groups of women. A 16-week HIIT intervention reduced resting heart rate but had no other effects on any of our selected CVD risk markers in women with PCOS. Participants allocated to the control group (PCOS Non-Ex) showed favorable improvements in body weight, systolic blood pressure, and circulating insulin levels after 16 weeks. Our findings highlight two important challenges in exercise training studies: low adherence to the prescribed exercise protocol in the intervention group and introduction of lifestyle changes in participants allocated to the control group.

## Figures and Tables

**Table 1:** Baseline characteristics of women with polycystic ovary syndrome (PCOS) and without PCOS (Non-PCOS).

		PCOS		Non-PCOS	*p*-Values
	*n*	Mean (SD)	*n*	Mean (SD)	
Age (years)	64	30 (5)	15	31 (6)	0.50
Body weight (kg)	64	85.1 (19.6)	15	81.2 (17.1)	0.48
Body mass index (kg/m^2^)	64	30.5 (6.5)	15	28.4 (5.6)	0.24
Systolic blood pressure (mmHg)	64	117.2 (9.8)	15	114.8 (8.4)	0.35
Diastolic blood pressure (mmHg)	64	75.2 (8.3)	15	76.1 (6.8)	0.70
Resting heart rate (beats/min)	64	64.7 (9.9)	15	57.1 (12.2)	**0.011**
MMP-9 (ng/mL) *	62	44.5 (27.0)	11	50.4 (27.6)	0.61
Total cholesterol (mmol/L)	64	4.4 (0.8)	13	4.3 (0.6)	0.62
HDL cholesterol (mmol/L)	64	1.3 (0.3)	13	1.4 (0.2)	0.65
LDL cholesterol (mmol/L)	63	2.6 (0.7)	13	2.5 (0.5)	0.60
Triglycerides (mmol/L)	63	1.1 (0.6)	13	0.9 (0.4)	0.23
Glucose (mmol/L)	63	5.0 (0.5)	13	4.8 (0.4)	0.40
Insulin (pmol/L)	64	117 (89)	12	84 (61)	0.28
HOMA-IR	63	4.5 (3.8)	12	3.0 (2.3)	0.24
Glucose area under the curve (mmol/L * min)	61	833 (199)	12	818 (151)	0.63
Glucose incremental area under the curve (mmol/L * min)	61	191 (147)	12	197 (106)	0.97
Insulin area under the curve (pmol/L * min)	59	69,652 (45,772)	6	62,069 (31,704)	0.71
Insulin incremental area under the curve (pmol/L * min)	59	46,803 (32,377)	6	43,001 (18,508)	0.79
Absolute FMD (mm)	33	0.19 (0.13)	10	0.28 (0.11)	0.064
FMD (%)	33	5.5 (4.1)	10	8.2 (3.9)	0.067
Resting Diameter (mm)	34	3.4 (0.3)	10	3.4 (0.2)	0.91
Peak Diameter (mm)	33	3.6 (0.3)	10	3.7 (0.2)	0.36
Time to peak (s)	33	44 (42)	10	19 (20)	0.073
IMT (mm)	20	0.56 (0.06)	4	0.57 (0.03)	0.96

* = three samples were excluded due to haemolysis. VO_2_peak, peak oxygen uptake; MMP-9, matrix metallopeptidase; HDL, high-density lipoprotein cholesterol; LDL, low-density lipoprotein cholesterol; HOMA-IR, homeostatic model assessment of insulin resistance; FMD, flow-mediated dilatation; IMT, carotid intima-media thickness. Statistically significant *p*-values are in bold.

**Table 2:** Effects of 16 weeks of high-intensity interval training (HIIT) or non-exercise (Non-Ex). Estimated mean difference from baseline values with 95% confidence interval (CI) from linear mixed models.

	PCOS HIIT		PCOS Non-Ex		Non-PCOS HIIT		PCOS HIIT vs. PCOS Non-Ex (Group x Time Interaction)		PCOS HIIT vs. Non-PCOS HIIT (Group x Time Interaction)	
	Estimate (95% CI)	*p*	Estimate (95% CI)	*p*	Estimate (95% CI)	*p*	Estimate (95% CI)	*p*	Estimate (95% CI)	*p*
Body weight (kg)	−0.44 (−1.36, 0.49)	0.35	−2.19 (−3.35, −1.03)	**<0.001**	−1.52 (−2.88, −0.17)	**0.028**	1.75 (0.27, 3.24)	**0.021**	−1.09 (−2.73, 0.55)	0.19
Body mass index (kg/m^2^)	−0.20 (−0.53, 0.13)	0.22	−0.79 (−1.20, −0.37)	**<0.001**	−0.54 (−1.02, −0.06)	**0.029**	0.58 (0.06, 1.1)	**0.031**	−0.34 (−0.92, 0.25)	0.25
Resting heart rate (beats/min)	−2.8 (−5.4, −0.2)	**0.037**	−1.1 (−4.2, 2.1)	0.50	−1.1 (−4.9, 2.8)	0.58	−1.7 (−5.7, 2.3)	0.40	1.7 (−2.9, 6.4)	0.46
Systolic blood pressure (mmHg)	−1.5 (−3.9, 0.8)	0.20	−4.8 (−7.6, −1.9)	**0.001**	−2.1 (−5.6, 1.3)	0.22	−3.2 (−6.8, 0.4)	0.078	−0.5(−4.7, 3.7)	0.81
Diastolic blood pressure (mmHg)	−1.7 (−4.0, 0.6)	0.14	−0.1 (−2.9, 2.6)	0.93	−0.7 (−4.1, 2.7)	0.68	−1.6 (−5.0, 1.8)	0.36	−1.3 (−2.8, 5.5)	0.53
Total cholesterol (mmol/L)	−0.07 (−0.25, 0.11)	0.42	−0.11 (−0.33, 0.11)	0.30	0.04 (−0.25, 0.32)	0.80	−0.04 (−0.32, 0.24)	0.77	0.12 (−0.22, 0.46)	0.48
HDL cholesterol (mmol/L)	−0.03 (−0.10, 0.04)	0.41	−0.12 (−0.20, −0.03)	**0.010**	−0.01 (−0.12, 0.11)	0.91	−0.09 (−0.20, 0.02)	0.12	0.02 (−0.11, 0.16)	0.74
LDL cholesterol (mmol/L)	−0.02 (−0.17, 0.13)	0.76	0.07 (−0.11, 0.26)	0.43	0.03 (−0.20, 0.27)	0.77	−0.10 (−0.33, 0.14)	0.41	0.06 (−0.22, 0.34)	0.67
Triglycerides (mmol/L)	−0.01 (−0.15, 0.16)	0.91	−0.14 (−0.33, 0.05)	0.15	0.03 (−0.22, 0.28)	0.82	−0.15 (−0.38, 0.09)	0.22	0.02 (−0.27, 0.32)	0.88
Glucose (mmol/L)	−0.11 (−0.25, 0.03)	0.11	−0.09 (−0.26, 0.08)	0.30	0.10 (−0.13, 0.32)	0.38	−0.03 (−0.24, 0.19)	0.81	0.20 (−0.06, 0.47)	0.13
Insulin (pmol/L)	−13.6 (−31.6, 4.4)	0.14	−25.1 (−47.2, −3.0)	**0.026**	−11.5 (−41.3, 18.3)	0.44	11.5 (−16.4, 39.4)	0.42	4.1 (−30.8, 39.1)	0.81
HOMA-IR	−0.66 (−1.48, 0.16)	0.11	−1.10 (−2.11, −0.10)	**0.032**	−0.35 (−1.72, 1.01)	0.61	0.44 (−0.83, 1.71)	0.49	0.39 (−1.21, 1.99)	0.63
Glucose area under the curve (mmol/L * min)	−34.7 (−84.0, 14.6)	0.17	−11.6 (−72.6, 49.4)	0.71	41.9 (−39.8, 123.6)	0.31	−23.1 (−98.8, 52.6)	0.55	70.4 (−25.6, 166.4)	0.15
Glucose incremental area under the curve (mmol/L * min)	−10.6 (−51.5, 30.4)	0.61	3.9 (−46.6, 54.4)	0.88	22.7 (−45.7, 91.1)	0.51	−14.5 (−76.7, 47.8)	0.65	26.1 (−54.1, 106.4)	0.52
Insulin area under the curve (pmol/L * min)	−3609.9 (−13,074.4, 5854.6)	0.45	−8898.2 (−20,769.0, 2972.7)	0.14	−12804.2 (−33,481.4, 7873.0)	0.22	−5288.3 (−20,193.7, 9617.1)	0.48	−8031.2 (−30,807.1, 14744.8)	0.48
Insulin incremental area under the curve (pmol/L * min)	−1240.4 (−8788.6, 6307.7)	0.74	−6499.5 (−15,949.9, 2950.9)	0.17	−7748.3 (−24,392.6, 8895.9)	0.35	−5259.0 (−17,060.2, 6542.1)	0.38	−5620.9 (−23,949.1, 12707.4)	0.54
MMP-9 (ng/mL)	−0.79 (−10.52, 8.94)	0.87	2.30 (−9.42, 14.02)	0.70	2.73 (−14.44, 19.90)	0.75	−3.09 (−17.30, 11.12)	0.67	4.48 (−15.49, 24.46)	0.66
IMT (mm)	−0.01 (−0.05, 0.03)	0.44	−0.00 (−0.08, 0.07)	0.96	0.03 (−0.02, 0.08)	0.14	0.01 (−0.07, 0.10)	0.73	0.04 (−0.02, 0.10)	0.15

PCOS, polycystic ovary syndrome; HIIT, high-intensity interval training; PCOS Non-Ex, PCOS non-exercise group; HDL, high-density lipoprotein cholesterol; LDL, low-density lipoprotein cholesterol; HOMA-IR, homeostatic model assessment of insulin resistance; VO_2_peak, peak oxygen uptake; MMP-9, matrix metallopeptidase; IMT, carotid intima-media thickness. Statistically significant *p*-values are in bold. The first four columns in the table are based on a linear mixed model with time and group allocation categorized in five categories: PCOS at baseline, PCOS Non-Ex at 16 weeks, PCOS HIIT at 16 weeks, Non-PCOS at baseline, and Non-PCOS at 16 weeks. The first three columns represent the within-group difference. The last two columns represent between-group differences. The last column is based on a linear mixed model with a three-category group variable (PCOS Non-Ex, PCOS HIIT, Non-PCOS HIIT) and time and their interaction as two fixed effects. The columns represent the interaction between the groups PCOS HIIT versus Non-PCOS HIIT, and time.

## Data Availability

Individual participant data from this study (after de-identification) will be available from the publication date of this manuscript. Proposal should be directed to the corresponding author Trine Moholdt (trine.moholdt@ntnu.no).

## Supplementary Materials

The following supporting information can be downloaded at: https://www.mdpi.com/article/10.3390/jcm11061626/s1, Figure S1: Participant CONSORT flow diagram.
