# Supplementary material for: Cardiovascular Health Does Not Change Following High-Intensity Interval Training in Women with Polycystic Ovary Syndrome
Source: J Clin Med. 2022 Mar 15;11(6):1626. doi: 10.3390/jcm11061626 (PMC8953804; doi:10.3390/jcm11061626)
Supplement: Supplementary file 1 [file jcm-11-01626-s001.zip › jcm-1601998-supplementary.pdf]

CONSORT 2010 Flow Diagram

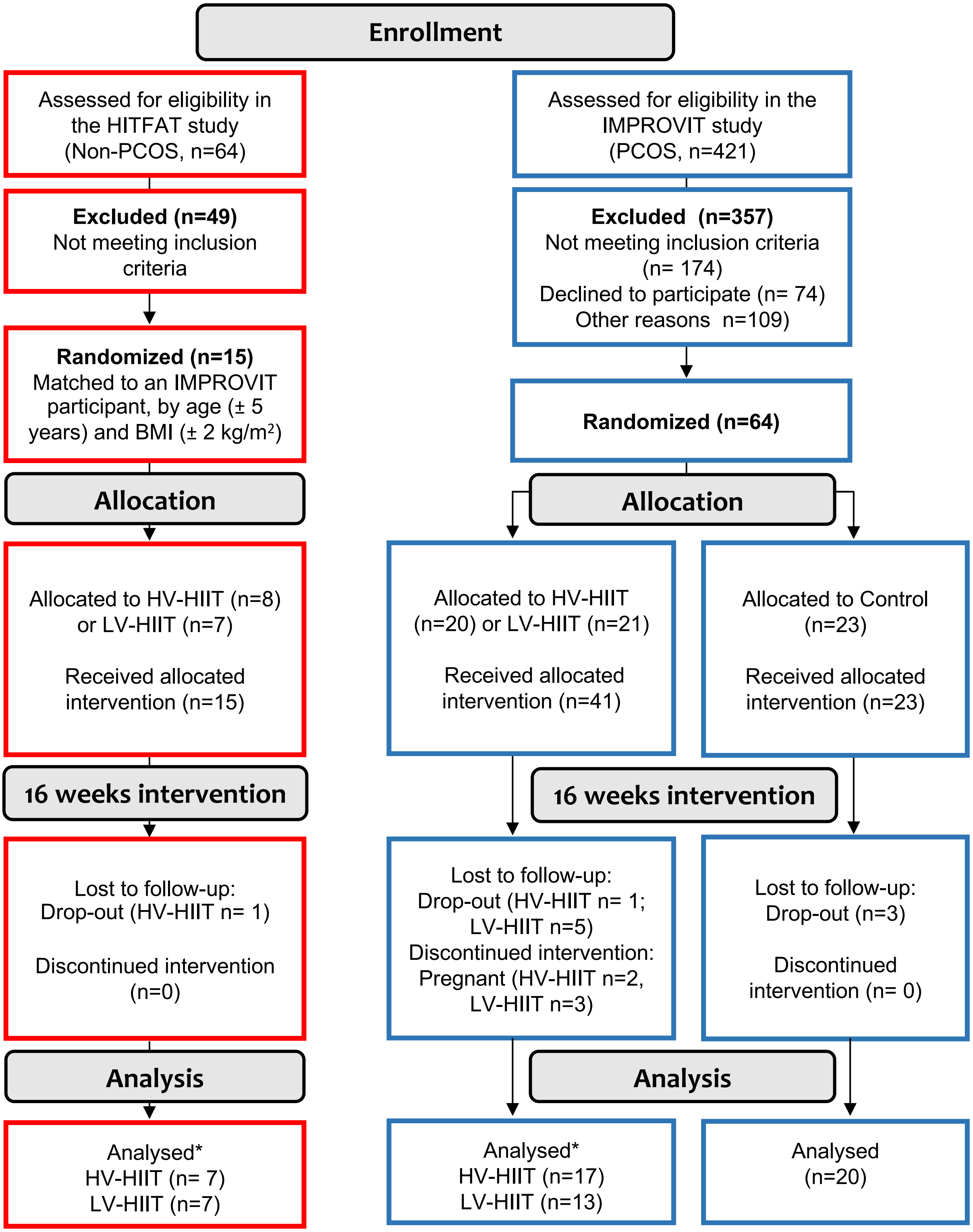

* Low-volume high-intensity interval training (LV-HIIT) and high-volume high-intensity interval training (HV-HIIT) were pooled in the analysis for both women with and without PCOS to improve statistical power.

Figure S1: Participant CONSORT flow diagram.
